# Quantification of cartilage and subchondral bone cysts on knee specimens based on a spectral photon-counting computed tomography

**DOI:** 10.1038/s41598-023-38238-y

**Published:** 2023-07-08

**Authors:** Célestin Garcelon, Juan Abascal, Cecile Olivier, Stéphanie Uk, Salim Si-Mohamed, Hang-Korng Ea, Philippe Douek, Francoise Peyrin, Christine Chappard

**Affiliations:** 1 Paris Cité University, CNRS, INSERM, B3OA UMR 7052 U1273, Paris, France; 2 University of Lyon, INSA-Lyon, CNRS, INSERM, CREATIS UMR 5220, U1206, Lyon, France; 3 Rheumatology Department, University Paris Cité, Paris, France

**Keywords:** Biophysics, Biomarkers, Rheumatology

## Abstract

Spectral photon-counting computed tomography (SPCCT) is a new technique with the capability to provide mono-energetic (monoE) images with high signal to noise ratio. We demonstrate the feasibility of SPCCT to characterize at the same time cartilage and subchondral bone cysts (SBCs) without contrast agent in osteoarthritis (OA). To achieve this goal, 10 human knee specimens (6 normal and 4 with OA) were imaged with a clinical prototype SPCCT. The monoE images at 60 keV with isotropic voxels of 250 × 250 × 250 µm^3^ were compared with monoE synchrotron radiation CT (SR micro-CT) images at 55 keV with isotropic voxels of 45 × 45 × 45 µm^3^ used as benchmark for cartilage segmentation. In the two OA knees with SBCs, the volume and density of SBCs were evaluated in SPCCT images. In 25 compartments (lateral tibial (LT), medial tibial, (MT), lateral femoral (LF), medial femoral and patella), the mean bias between SPCCT and SR micro-CT analyses were 101 ± 272 mm^3^ for cartilage volume and 0.33 mm ± 0.18 for mean cartilage thickness. Between normal and OA knees, mean cartilage thicknesses were found statistically different (0.005 < *p* < 0.04) for LT, MT and LF compartments. The 2 OA knees displayed different SBCs profiles in terms of volume, density, and distribution according to size and location. SPCCT with fast acquisitions is able to characterize cartilage morphology and SBCs. SPCCT can be used potentially as a new tool in clinical studies in OA.

## Introduction

Imaging modalities which contribute to a better understanding of osteoarthritis (OA) are radiographs, magnetic resonance imaging (MRI), X-ray computed tomography (CT), ultrasound, dual energy absorptiometry and positron emission tomography. Among these modalities, MRI and CT provide three-dimensional images, MRI is classically used in clinical routine to visualize cartilage, joint effusion, ligaments, tendons, meniscus, osteophytes, and bone marrow lesions or subchondral bone cysts (SBCs). In research, semi-quantitative scoring and quantitative assessment of tissue dimension (volume, thickness) are possible on MRI images^1^. Three dimensional sequences recently developed allow thin continuous slices and reduced the partial volume effect^2^. Compositional MRI based on sequences such as T2, T2*, and T1 ρ, relaxation times and glycosaminoglycans chemical exchange saturation transfert (gagCEST) and sodium imaging can give additional information about cartilage degeneration without contrast agent^1^. Using contrast agent, delayed gadolinium-enhanced MRI of cartilage (dGEMRIC) can give information about the proteoglycans content^1^. Subchondral bone cysts are associated with an increased risk of knee replacement^3^, and may predict symptoms^4^. Based on a micro-CT study ex-vivo, they are formed to concentric (re)arrangement of trabeculae around an enlarged marrow space^5^.

CT is known to enable a good visualization of bone and calcified tissue and has been proposed to evaluate calcium deposits which play a role in initiation and disease progression^6^. Dual energy CT provides good diagnostic accuracy for the detection of monosodium urate crystals^7^ but requires further investigation in case of calcium pyrophosphate deposition diseases^8^.

The progress of OA management requires the development of non-invasive diagnostic methods able to quantify cartilage and subchondral bone changes^9^. Recently, spectral photon-counting computed tomography (SPCCT) has been introduced in the clinical field. They are characterized by new detectors, i.e., energy-resolving photon-counting detector, that simultaneously count photons and resolve their energy^10^. In living patients, SPCCT has, for instance, been already used to characterize kidney stones^11^, coronary artery calcium scoring^12^, coronary stent^13^, lung cancers^14^, brain^15^ and anatomical structures of temporal bone and wrist^16,17^. SPCCT extends the dual energy CT approach by a multispectral approach^18^.

Photon-counting detectors compared to energy-integrating integrator (EI) detectors usually found in conventional clinical CT were demonstrated to reduce the image noise and the dose in describing the fine anatomy of cadaveric wrists^19^, and for the quantitative assessment of trabecular bone micro-architecture on vertebral phantoms^20^. The Cadmium Telluride (CdTe) detectors are able to convert energy of an X-ray into an electrical signal with a magnitude proportional to the incident photon’s energy. Each individual pulse is counted separately thanks to fast electronics with an equal contribution whatever its energy.

The signal generated by a single X-ray photon is short enough to decay before the next photon arrival, then the incoming X-ray are compared to a threshold voltage, each threshold determining separated energy bins^10,21^.

One of the main application of SPCCT is in K-edge imaging, the principle being that the linear attenuation coefficient of contrast agents such as iodine, gadolinium, gold or bismuth presents discontinuity when crossing their K-ray energy^22^. K-edge imaging was suggested to give potential additional information on cartilage glycosaminoglycans content in excised osteoarthritic knees^23^. Using a dual contrast agent method, it was possible to assess the proteoglycans and water content in human knee cartilage^24^. This approach was confirmed in a study using SPCCT arthrography in mono-iodo-acetate injected knees of living pigs^25^. Moreover, SPCCT is able to provide material decomposition maps since the attenuation of each material being dependent of the energy, it becomes possible to obtain biochemical information visualized as color overlay images^10^. Based on this method, the differentiating of calcium pyrophosphate and hydroxyapatite deposits were suggested in *in-vitro* studies^26,27^.

In addition to the improvement of the compromise between the image quality and the dose delivery, photon-counting detectors are able to exploit spectral information and generate virtual mono-energetic images with a potential to improve the contrast between tissues with intrinsic low attenuation. In a previous study, we have demonstrated that virtual mono-energetic images at 60 keV based on SPCCT scanners were the best choice for both bone and cartilage visualization^28^.

We present, as a proof of concept, results of the quantitative assessment of cartilage without any contrast agent, and subchondral bone cysts obtained from virtual mono-energetic images of knees specimens acquired with a whole body helical SPCCT. Cartilage assessment will be compared with high resolution mono-energetic synchrotron radiation CT images with phase contrast (which improve the soft tissue contrast) used as benchmark.

## Methods

### Knee specimens

Ten knee specimens with all soft tissues (ligaments, muscles, skin), 6 normal and 4 with OA were taken from the Institut d’Anatomie Paris from 8 females and 2 males (mean age: 85.4 ± 13.1) selected from a set of 30 knees. The Kellgren Lawrence (KL) classification was estimated from frontal views of high resolution peripheral computed tomography scans. The four OA knees were characterized by KL ≥ 2 and two of them present subchondral bone cysts. No additional information was available regarding cause of death, previous illnesses, or any medical treatments of these subjects except for an absence of hepatitis and human immunodeficiency virus. The subjects willed their body to science in accordance with the Declaration of Helsinki and were anonymous. All methods were performed in accordance with the relevant guidelines and regulations. The protocol was approved by the French Ministry of Higher Education and Research (CODECOH number DC-2019-3422). The study was approved by the ethics committee of the Institut of Anatomy of Paris. Informed consent was obtained from all participants. All specimens were stored at −20 °C. SPCCT acquisitions were performed on frozen knees and SR micro-CT acquisitions after thawing.

### Spectral photo-counting acquisitions

The knee specimens were imaged on a clinical SPCCT system prototype (Philips Healthcare, Harifa) installed at Lyon (CERMEP), France. The knee specimens were acquired in helical mode, at 120 kVp with five energy thresholds in the range of 30–120 keV (30, 51, 62, 72, 81) and 165 mAs, a field of view of 180 × 180 × 80 mm^3^ (matrix size: 720 × 720 × 320) and a pitch of 1.027. For each volumetric scan, CDTI_vol_ was 16.6 mGy. The duration scan was less than 5 min. Then, virtual mono-energetic images were obtained by combining the decomposed material maps reconstructed from an adapted Feldkamp algorithm. The isotropic voxel size was 250 × 250 × 250 μm^3^ in the reconstructed images. For comparison, we used mono-energetic synchrotron radiation CT (SR micro-CT) images at 55 keV with phase contrast (ESRF, Grenoble beamline ID 17) acquired with a cubic voxel size of 45 μm.

### Image segmentation protocol

**Figure 1:**
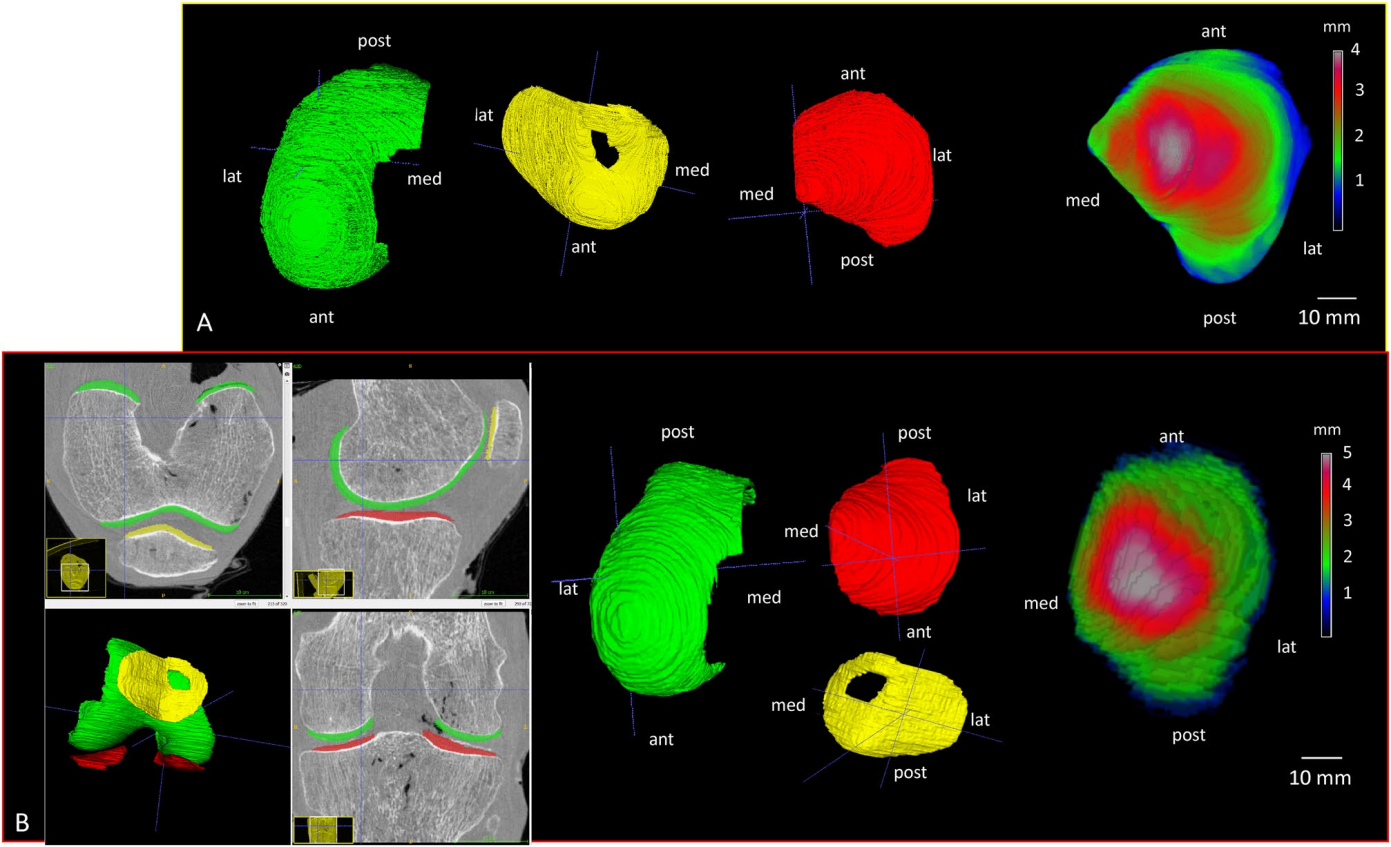
Examples of cartilage segmentations for both SR micro-CT images (**A**) and SPCCT (**B**), on lateral femur, patella and lateral tibia, the cartilage segmentation was performed manually, the contrast being sufficient enough to segment the cartilage thanks to the three orthogonal directions. On the right, examples of mapping of the lateral tibia cartilage, on SR micro-CT image (top) and on SPCCT image (bottom). In blue is represented the lowest values of local cartilage thickness and in white the highest values. A normal knee is presented.

**Figure 2:**
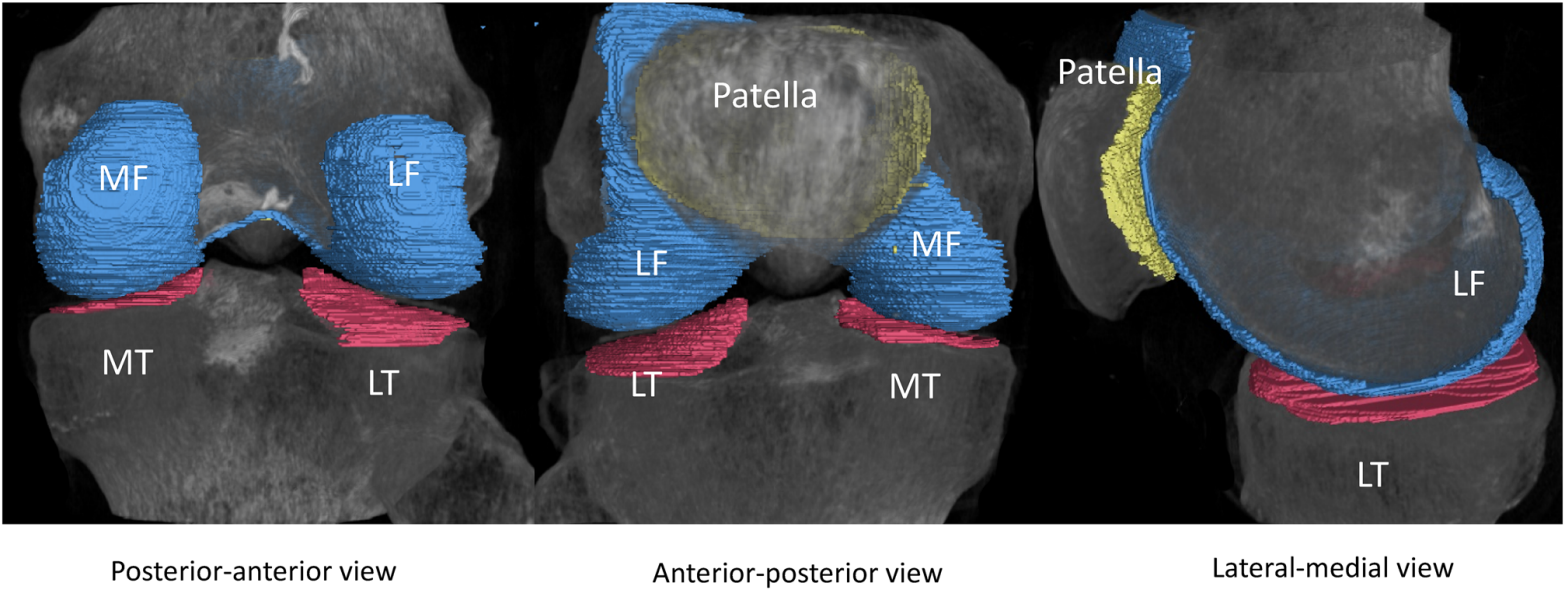
Examples of cartilage segmentation for SR micro-CT images (top) and SPCCT images (bottom) with from right to left: postero-anterior views, antero-posterior views and lateral views. Each compartment is analyzed separately: tibia lateral (red), femoral lateral (blue), tibia medial (red), femoral medial (blue) and patella (yellow). A normal knee is presented.

**Figure 3:**
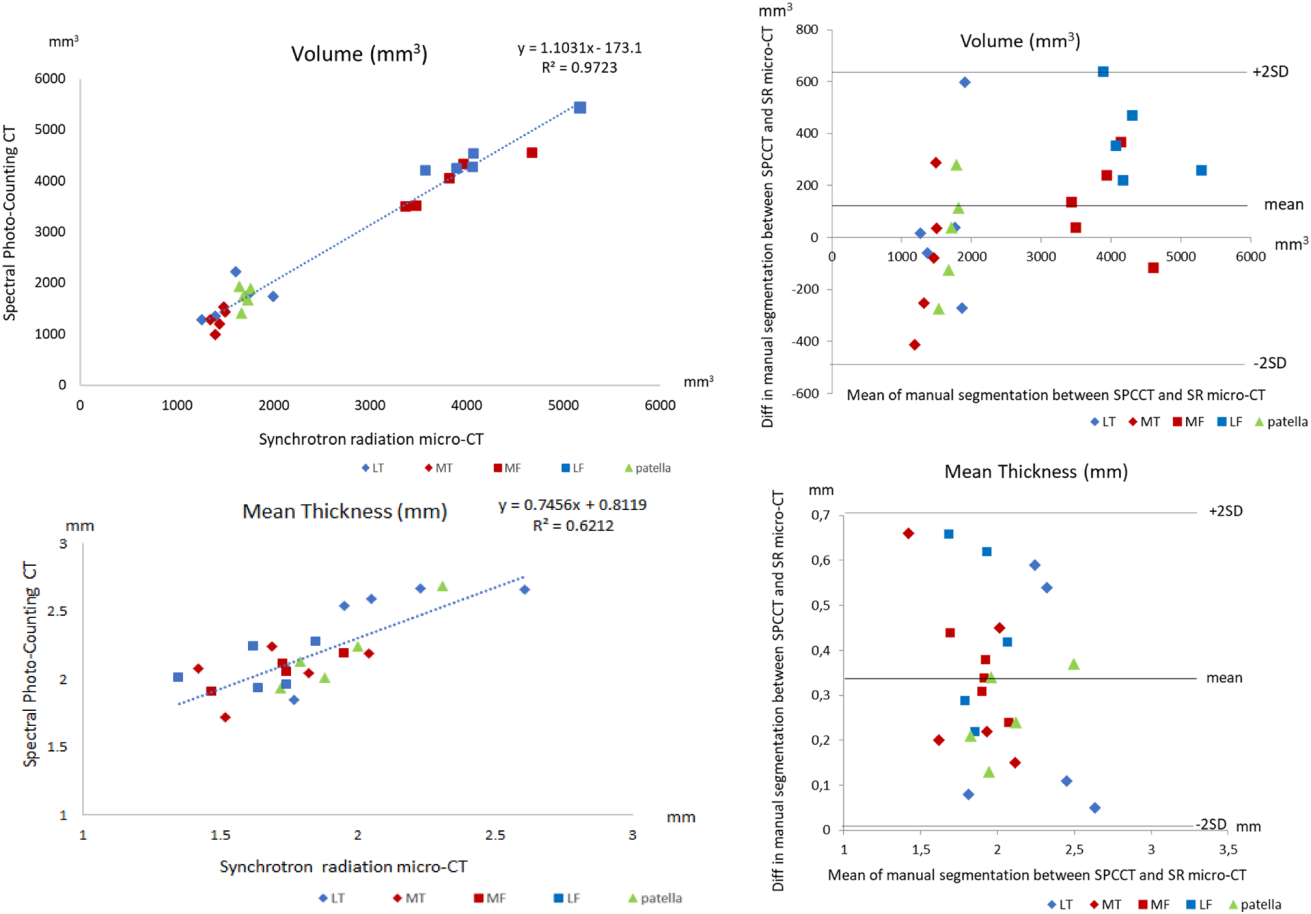
Regression analyses (left) and Bland et Altman plots (right) of cartilage maps comparing SPCCT and SR micro-CT used as benchmark for volume in mm^3^ (top graphs) and mean thickness in mm (bottom graphs). Lateral tibia (LT) and lateral femur are presented in blue, medial tibia and medial femur in red, and patella in green.

The 3D segmentations of cartilage were performed manually with itk-SNAP 3.8.0 for both SPCCTand SR micro-CT images by a rheumatologist (CC) expert in quantitative image analysis for osteo-articular diseases for 20 years (Fig. 1). For medial and lateral compartments, cartilage of tibia and femur were separately labeled, which gives us four compartments per knee: lateral tibia (LT), medial tibia (MT), lateral femur (LF), medial femur (MF) and finally the patella which corresponds to a single label (Fig. 2). To smooth the surface, a median filter of one voxel was applied in SPCCT and one of 5 voxels on SR micro-CT images. Then, the measurements of the volume (mm^3^) and thickness (mm) of the cartilage segmented mask were obtained with CTAn (Skyscan Bruker, Belgium). The thickness measurements were based on the sphere method^29^. Non parametric Wilcoxon paired tests were conducted to compare cartilage volume and thickness provided by both modalities (SPCCT and SR micro-CT). An example of cartilage segmentation for both acquisition methods are presented in Fig. 3. Furthermore, regression analyses with determination coefficients and Bland and Altman plots for volume and cartilage thickness were conducted to examine relationships and offsets between SPCCT and SR micro-CT (Fig. 3). The cartilage segmentation was performed two times on SPCCT images by the same operator for the whole data sets and we calculated for cartilage volume and thickness the Root Mean Standard Deviation in mm (RMSSD, mm) and the Root Mean Square Coefficient of Variation in % (RMSCV, %)^30^.

**Figure 4:**
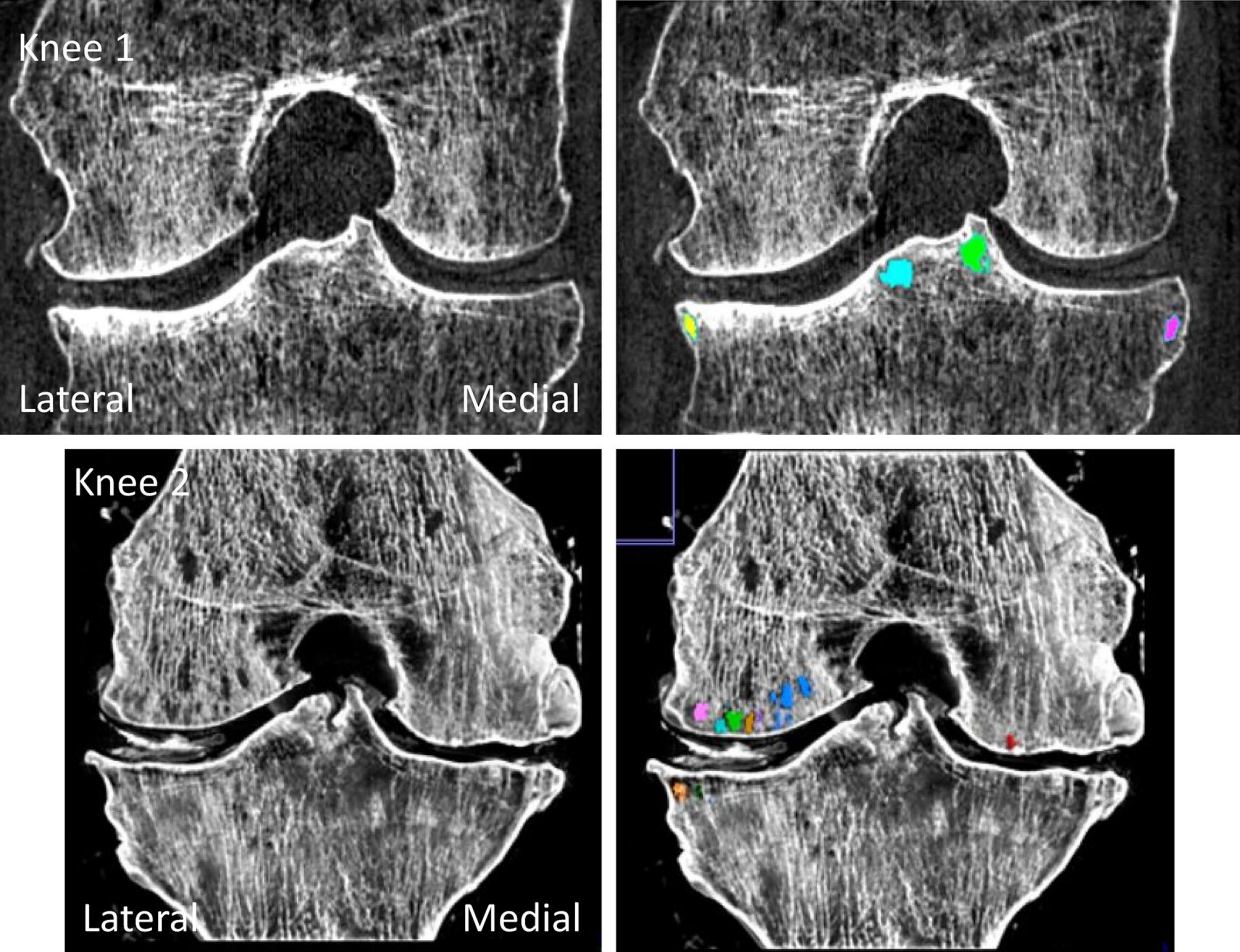
Examples of subchondral bone cysts selected in the first 10 mm under the subchondral plate for the 2 osteoarthritis knees (knee 1 and knee 2). The subchondral bone cysts segmentation is based on a region growing process which permits quantitative measurements such as volume and density.

On SPCCT images, 3D segmentations of subchondral bone cysts (SBCs) were performed with AVIZO 9.0 on 2 OA knees. A subchondral region of 10 mm depth was isolated, each SBCs were pointed out by operator for initialization and an automatic growing region process was applied. Then, SBCs volume (mm^3^) were measured and density (1/mm^3^) were calculated and corresponds to the number of SBCs over the total volume of the subchondral region. All bone cysts with a volume inferior to 3 mm^3^ were excluded for final results. Analyses were performed with the same labeling as the cartilage (Fig. 4).

## Results

### Cartilage

The mean cartilage thickness with their standard deviation results for each compartment are presented in Table 1. The minimum and maximum values of the lateral tibia are (1.77–2.61 mm) for SR micro-CT and (1.85–2.67 mm) for SPCCT, and for the medial tibia (1.42–2.04) and (1.72–2.24), respectively. For femoral compartments in lateral position, the minimum and maximum values are (1.35–1.85) for SR micro-CT and (1.93–2.27) for SPCCT and in medial position (1.47–1.95) and (1.91–2.19), respectively. The cartilage thickness minimum and maximum values of the patella are (1.72–2.31) for SR micro-CT and (1.93–2.68) for SPCCT.

**Table 1:** Mean cartilage thickness and standard deviation assessed from 3D maps obtained on SR micro-CT and SPCCT images.

	Cartilage Thickness (mm) mean ± SD		Wilcoxon test
	SR micro-CT	SPCCT	*p*
Lateral femoral	1.64 ± 0.18	2.08 ± 0.16	0.004
Medial femoral	1.73 ± 0.17	2.07 ± 0.10	0.008
Lateral tibia	2.21 ± 0.32	2.46 ± 0.34	ns
Medial tibia	1.72 ± 0.24	2.05 ± 0.20	0.03
Patella	1.94 ± 0.23	2.20 ± 0.29	ns

The comparison was made with a Wilcoxon test.

No statistical differences were found between SPCCT and SR micro-CT cartilage segmentations for volume, standard deviation, minimum and maximum. We found statistical differences between SR micro-CT and SPCCT for femur (0.004 < *p* < 0.008) and for medial tibia (*p* = 0.03).

For cartilage volume the RMSSD was 150 mm^3^ and RMSCV was 5.67% and for cartilage thickness was the RMSSD was 0.064 mm and the RMSCV% was 2.94%.

From the 25 compartments, the determination coefficients are 0.97 and 0.62 for cartilage volume and thickness, respectively. The mean biases obtained based on the Bland and Altman methods are 101mm^3^ ± 272 for volume and 0.33 mm ± 0.18 for mean thickness (Fig. 3). The mean bias is about 6 times lower than the mean thickness measured.

A comparison of the six normal knees and the four OA knees are displayed in Table 2. We found significant differences for lateral femur, lateral tibia and medial tibia especially for volume and cartilage thickness with 0.005 < *p* < 0.04.

**Table 2:** Comparisons of mean ± Standard Deviation for quantitative parameters such as volume, mean thickness, standard deviation an maximum corresponding to cartilage maps in different compartments (Lateral and Medial Femur, Lateral and Medial Tibia, and patella) between six normal and four osteoarthritis knees.

	Lateral femur	Medial femur	Lateral tibia	Medial tibia	Patella
Volume (mm^3^)					
Normal	4678 ± 739	4057 ± 600	1793 ± 374	1247 ± 175	1682 ± 183
OA	3288 ± 897	2823 ± 1636	1025 ± 268	746 ± 411	1607 ± 470
*p*	0.04	ns	0.005	0.04	ns
Mean thickness (mm)					
Normal	2.12 ± 0.15	2.06 ± 0.08	2.63 ± 0.29	2.10 ± 0.21	2.21 ± 0.28
OA	1.79 ± 0.14	1.75 ± 0.42	1.94 ± 0.34	1.62 ± 0.31	1.83 ± 0.42
*p*	0.009	ns	0.01	0.01	ns
Standard deviation thickness (mm)					
Normal	0.52 ± 0.05	0.51 ± 0.03	0.79 ± 0.15	0.53 ± 0.13	0.60 ± 0.16
OA	0.50 ± 0.09	0.47 ± 0.09	0.51 ± 0.16	0.40 ± 0.08	0.40 ± 0.10
*p*	ns	ns	0.02	ns	0.01
Maximum thickness (mm)					
Normal	3.58 ± 0.26	3.33 ± 0.20	4.1 ± 0.6	3.17 ± 0.58	3.5 ± 0.52
OA	5.87 ± 0.63	3.25 ± 0.41	3.0 ± 0.29	2.62 ± 0.48	2.87 ± 0.63
*p*	0.03	ns	0.008	ns	ns

The Wilcoxon test was used for comparison.

### Subchondral bone cysts

**Figure 5:**
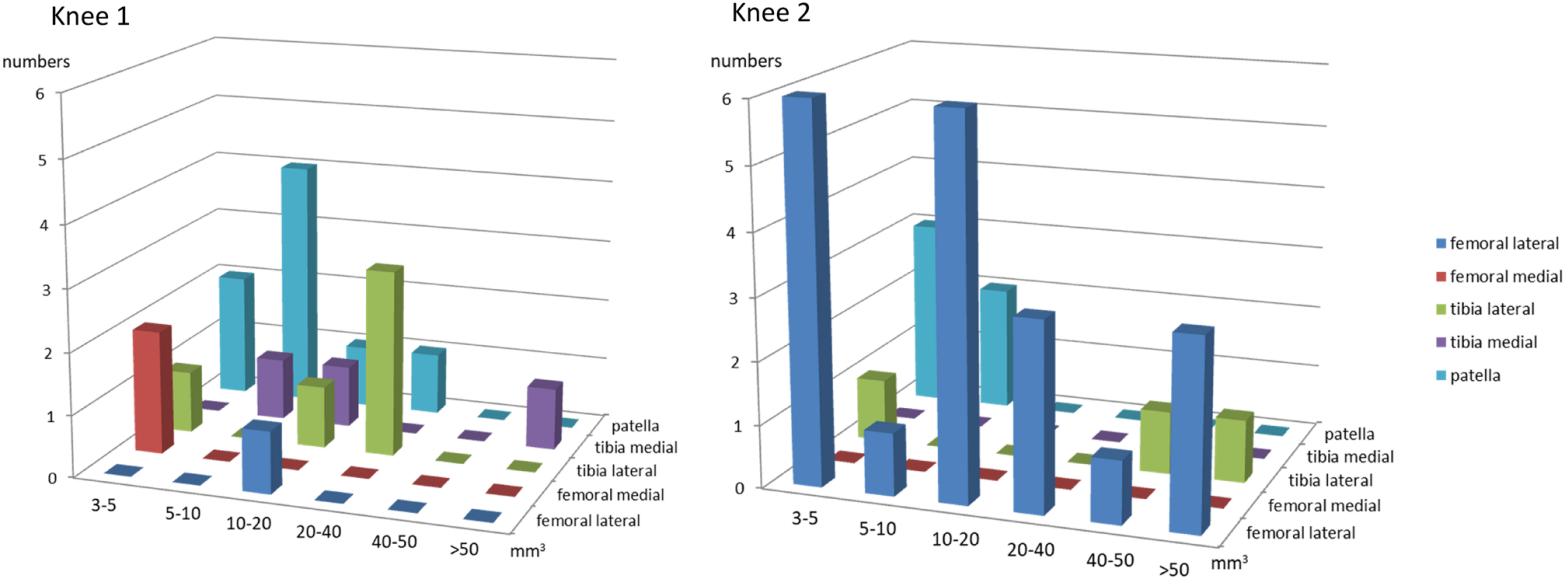
Subchondral bone cysts distribution according to size and location for the two osteoarthritis knees. Different patterns are clearly individualized for knee 1 and knee2.

The median SBCs volume and mean SBCs density results are shown in Table 3. At femur SBCs volumes varied from 3.7 to 16.2 mm^3^ for knee 1 and 3.3–85.8 mm^3^ for knee 2. At tibia, SBCs volume varied from 3.0 to 86.8 mm^3^ for knee 1 and 3.3–79.4 for knee 2. At patella, SBCs volume varied from 4.3 to 33.9 mm^3^ for knee 1 and 3.1–6.8 mm^3^ for knee 2. Subchondral bone cysts densities varied from 0.03 to 5.6 × 10^–3^ mm^−3^ for knee 1 and 2.5–20.6 × 10^–3^ mm^−3^ for knee 2. The SBCs distributions according to size are displayed in Fig. 5 for both knee 1 and knee 2. For knee 1, SBCs are predominating with small cysts under 40 mm^3^ in patella and lateral tibia and for knee 2, small cysts about 5–10 mm^3^ are present at the patella and lateral femur with also large pores > 10mm^3^ at lateral femur.

**Table 3:** Median bone cysts volume (mm^3^) and Mean Bone cysts density (10^–3^*1/mm^3^) for each subregions: femoral lateral, femoral medial, tibial lateral, tibial medial and patella in the two osteoarthritic knees.

	Median Bone cysts volume (mm^3^) (minimum–maximum)		Mean Bone cysts density (10^–3^*1/mm^3^)	
	Knee 1	Knee 2	Knee 1	Knee 2
Femoral lateral	16.2	12.0 (3.3–85.8)	0.5	20.6
Femoral medial	4.0 (3.7–4.3)	–	0.3	–
Tibia lateral	20.2 (3.0–25.2)	45.5 (3.7–79.4)	4.2	6.6
Tibia medial	12.6 (8.4–86.8)	–	3.5	–
Patella	9.2 (4.3–33.9)	4.8 (3.1–6.8)	5.6	2.5

## Discussion

The SPCCT prototype used in the present study is based on a clinical CT scanner and displayed all its characteristics with the potential to be used in a short period of time in clinics. Performances were already tested in living patients for brain, lung, heart, and to describe the anatomy of temporal bone and wrist^11–17^, but never for osteo-articular diseases. Our results suggest that SPCCT has the potential to provide biomarkers for OA characterization for both cartilage, and bone.

In a previous study, we have provided a proof of concept that SPCCT imaging using virtual mono-energetic images could improve the cartilage, and SBCs detectability compared to conventional CT based on EI detectors^28^. This SPCCT scanner uses a standard polychromatic source and photon counting detectors able to discriminate photons according to their energy and to separate them into several bins^10^. Its intrinsic spatial resolution allows small voxel sizes about 250 microns, which is twice as better than usual CT, the geometry efficiency being due to a lack of septa inside the detector^10^. The other advantages are the fast acquisition times less than 5 min, improved signal to noise ratio due to the exclusion of electronic noise from photon and pulse counts^10,20,21^. Low dose radiation estimates to be 30–60% lower compared to conventional CT^10^. It was confirmed compared to conventional CT,^31^ and in living patients^14,16^.

Compared to^28^, we have used a helical acquisition instead of a 2D slices acquisition and soft tissue around knee specimens were preserved to be close as possible to the clinical situation and did not impair the image quality and cartilage detection. Exploiting the virtual mono-energetic images is new and permits to increase the soft tissue contrast and is intrinsically able to provide a signature of each tissue. In our previous study, the contrast of the mono-energetic images at 60 keV between cartilage and meniscus was good and sufficient to visualize details in cartilage such as defects compared to SR micro-CT^28^. In the present study, the contrast was good enough to carry out a manual segmentation of the cartilage.

Articular cartilage loss is an acceptable measure of structural disease progression^1,2^. Isotropic 3D images and high resolution are both important features to quantify cartilage because this one at sub-regional level can show simultaneously thinning and thickening areas^1^. The cartilage thinning is usually assessed on 3D MRI images which present as advantages a good contrast, however obtaining isotropic 3D acquisitions at high spatial resolution is still challenging and necessitates high field ≥ 3 T with as disadvantage a low SNR compared to 2D acquisitions with large slice thicknesses^1,2^. Moreover, the best isotropic voxel size that can be achieved with a 3D MRI is 0.5*0.5*0.5 mm^2^. In the present study, the benchmark was SR micro-CT at 45 µm of resolution because it is the gold standard for mono-energetic X-ray tomography^32^. We obtained mean cartilage thickness measurements around 2 mm on SPCCT images in the same order of magnitude than those described in isotropic T1 ρ MRI images^33^ and in a study based on sharp needle measurements on fresh cadavers^34^. We have found good correlation for volume and thickness of cartilage and small non-proportional bias for cartilage volume measurements nevertheless, the cartilage thickness bias was 6 times less than the mean value. Cartilage maps of the femur are statistically different between SR micro-CT and SPCCT. The main reason is the partial volume effect, indeed, the increase in thickness due to the increase in voxel size is a well-documented phenomenon^35,36^. It is also due to a very complex structure compared to the tibia and patella which facilitates error measurements. Finally, thin peripheral zones are more visible on SR micro-CT and therefore accounted for, which explains the lower average values compared to SPCCT. However, we emphasize that central areas are more pertinent to evaluate than peripheral areas in OA. The freezing which may increase the cartilage volume and thickness about 10% (0.17–0.22 mm) could also explain the increase of volume and mean thickness in SPCCT data. Using the 3 orthogonal views was very helpful to complete this segmentation, since when details were lacking in one direction, one of the orthogonal views permitted to correct the segmentation. For both SR micro-CT and SPCCT, we used isotropic voxels which are essential for allowing quantitative analysis not sensitive to position errors. In future, for this process, artificial intelligence, as it was previously demonstrated for MRI cartilage segmentation^37^, could be implemented.

Clinical CT was previously used to explore subchondral tibial bone cysts, with a semi-automatic region growing guide by the observer^38^. In the present study, using a similar segmentation method, we found very different profiles in terms of volume and density from one knee to the other. Subchondral bone cysts were identified to be associated to knee pain in existing radiographic OA and that SBCs development may vary from patient to patient^3,4^ and influence biomechanical properties with increasing cartilage and intra-osseous stress^38^. Consequently, it is useful to have tools to assess them more accurately. We have excluded in the present study, the SBCs inferior to 3 mm^3^ which are not clinically pertinent and can be potentially considered as noise. Indeed, the cartilage stress has been found proportional to the SBCs size^39^. The subchondral bone plate was not assessed because the spatial resolution was not sufficient. In a previous study with micro-CT images about 10 microns^40^, we have evaluated the subchondral plate thickness found about 0.5 mm which corresponds to only 2 voxels at the present spatial resolution.

This study has some limitations. We examined very few knee specimens among them only 2 osteoarthritis specimens presented SBCs, it is due to the allocation of a limited beam time at the ESRF and a long acquisition time. We did not use MRI, which is the clinical reference for cartilage analysis. The main reason is that we adopted a multi-scale approach, the highest resolution being the reference for the lowest resolution. In addition, SR micro-CT is the reference for mono-energetic X-rays tomography and phase contrast increases the distinction between soft tissues. Regarding image analyses, manual cartilage segmentation is a tedious post-processing which necessitates a good expertise in anatomy of the operator. One more limitation of our study is that SBCs can be due to OA but it cannot be completely excluded that they could be related to local bone degradation of the cadaveric knees. However, our approach is a proof of concept of the methodology and this ambiguity will be avoided in living patients. The present study is a proof of concept and must be confirmed in vivo in a large population.

In conclusion, we have shown for the first time that SPCCT without using contrast agent allows direct visualization of cartilage and bone integrity, which are essential for the characterization of OA diseases affecting the whole joint. In addition, these results suggest that quantitative measurements of cartilage (volume and thickness) and subchondral bone cysts (volume and density) from mono-energetic SPCCT images could lead to new OA biomarkers. In the near future, this new technique could be proposed to patients to improve the knowledge of natural course and treatment responses in OA.

## Data Availability

The data sets used and/or analyzed during the current study are available from the corresponding author on reasonable request.
